# Bored Rotten: Interactions Between the Coffee Berry Borer and Coffee Fruit Rot

**DOI:** 10.3390/insects16040342

**Published:** 2025-03-25

**Authors:** Paul Bayman, Luz M. Serrato-Diaz

**Affiliations:** 1 Department of Biology, University of Puerto Rico–Río Piedras, P.O. Box 23360, San Juan, PR 00931-3360, USA; 2 Tropical Agriculture Research Station (TARS), U.S. Department of Agriculture-Agricultural Research Service, 2200 P.A. Campos Ave., Ste. 201, Mayagüez, PR 00680-5470, USA

**Keywords:** *Beauveria*, coffee berry disease, coffee fruit rot, *Colletotrichum*, *Fusarium*, *Hypothenemus hampei*

## Abstract

The coffee berry borer (CBB) is the most destructive pest of coffee worldwide, with damages exceeding $500 M a year and affecting the livelihood of 25 million farmers. Coffee fruit rot (CFR) is described as an anthracnose disease; it can cause up to 80% loss of the crop on susceptible cultivars when conditions favor it. These two serious threats to coffee production have been studied separately, but a link between them was not shown until recently. Several recent studies show that CBB damage is associated with a higher incidence of fruit rot; CBBs carry *Colletotrichum* and *Fusarium* fungi, also found in rotted fruits, and can transmit disease to coffee fruits as they bore into them. Previous studies on the relationship between CBB and *Fusarium* did not take into account that *Fusarium* is involved in coffee fruit rot, so these recent findings shed new light on the relationship. Here we discuss this relationship and its implications, both ecological and practical.

## Simple Summary

The coffee berry borer and coffee fruit rot are serious problems for coffee plants and growers. Until now they have been treated separately, but recent studies have shown they are closely linked. In this review, we consider the implications of new data for the coffee plant and the coffee berry borer. This synthesis provides additional incentives to control the coffee berry borer, since it will also help to reduce coffee fruit rot.

## 1. Coffee Fruit Rot and Coffee Berry Disease

The coffee berry borer (CBB) *Hypothenemus hampei* (Coleoptera: Scolytidae) is the most damaging insect pest of coffee worldwide [1]. CBB infestation affects both coffee yield and quality, and causes losses estimated at $500 M per year [2].

**Figure 1:**
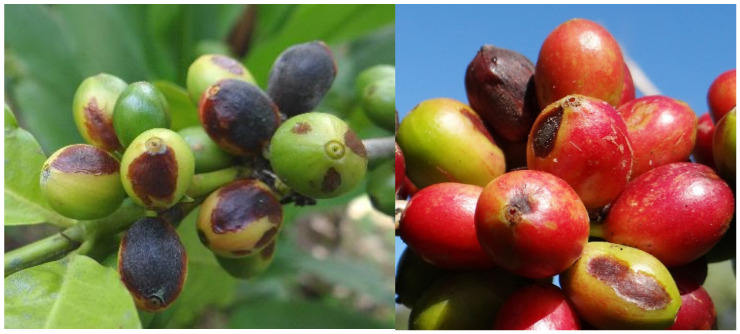
Coffee fruit rot lesions on green, yellow, and red coffee fruits. Coffee berry borer holes are visible in several fruits (**right**); (**left**) Ciales, PR, elevation 340 masl; (**right**) Adjuntas, PR, elevation 575 masl.

*H. hampei* needs no introduction for readers of this Special Issue, but coffee fruit rot (CFR) is less familiar. Coffee fruits may develop discolored sunken lesions (Figure 1). The lesions are more common on ripening (yellow or red) fruits but may also be observed on green fruits [1,3,4,5,6]. Fruits with lesions can become dry and mummified. The disease may lead to fruit abscission and affect coffee yield and quality, often causing losses of 20–30%, and much higher in susceptible cultivars when rainfall is high [1,7]. At high altitudes, and in the absence of effective control, losses frequently exceeded 80% [8]. It is partly responsible for the decrease in Africa’s share of world coffee production over the last fifty years [9].

There has been considerable confusion about the name of the disease and the identity of the pathogen. Fungal rot of coffee fruits was first described in Kenya in 1922 [3]. The disease was named coffee berry disease (CBD), and the causal agent was a close relative of the widespread and well-known species *Colletotrichum gloeosporioides*, later described as *C. coffeanum* var. *virulans* [4]. The distribution of *C. coffeanum* was reported to be limited to East Africa, which is odd considering that the species was described based on type material from Brazil [10]. The pathogen spread to all major coffee-producing areas in Africa in the following decades [11,12] but, as far as is known, is still limited to Africa [13].

The name was changed to *C. kahawae* [14] and then, based on DNA sequence-based phylogenies, to *C. kahawae* ssp. *kahawae* [15]. *C. kahawae* ssp. *kahawae* was proposed to have arisen from a more generalist pathogen, *C. kahawae* ssp. *cigarro*, following a host jump to coffee fruits [16]. However, there was resistance to reducing the coffee fruit pathogen to the level of subspecies, partly because *C. kahawae* is subject to quarantine [17] and changing the name could cause confusion for plant protection enforcement [10]. Furthermore, the high diversity of *Colletotrichum* species in coffee farms, both on coffee plants and on other substrates, increases the risk of misidentification [18]. Mistaken identification of *C. kahawae* can potentially compromise the phytosanitary status of the crop and country. For example, an isolate from a tree tomato in Colombia was identified as *C. kahawae* by morphology and DNA sequences [19]. Because the presence of *C. kahawae* ssp. *kahawae* could be grounds for quarantine restrictions, additional sequencing was conducted to demonstrate that this fungus was *C. kahawae* ssp. *ciggaro* [20]. Extensive data of several types supported maintaining *C. kahawae* as a separate species [13].

The claim that *C. kahawae* is the only species that can rot green coffee fruits, and only at high elevations in Africa, has become entrenched in the literature [1]. This is clearly not true (see Figure 1). Similarly, the name ‘coffee berry disease’ is only applied to Africa. For example, the *Compendium of Coffee Diseases and Pests* states: “Coffee berry disease is confined to date to the African continent…” and does not mention that other species of *Colletotrichum* cause anthracnose on green coffee fruits elsewhere [1]. Giving the same disease a different name because it occurs in a different place and involves a different (but very closely related) pathogen seems unreasonable; it leads to further confusion and proliferation of names.

Also, species’ names change because the taxonomy of the group is still in flux [10,13]. The current state of fungal taxonomy recognizes many cryptic species that are similar morphologically but are distinguished by differences in DNA sequences. This means that comparisons with papers written before DNA sequencing was widely available are limited, because it is unclear what the fungi they describe would be called today. Since the fungi they worked with are usually not available in culture collections, there is often no way to compare pathogens among studies. As more sequences are added to provide higher resolving power to phylogenetic analyses, it is expected that many existing names will change in the next few years.

Here, we follow what has become standard usage: we use **coffee berry disease** (CBD) to refer to the disease in Africa caused by *C. kahawae* and **coffee fruit rot** (CFR) to refer to the same disease elsewhere caused by other *Colletotrichum* species [6].

## 2. Coffee Fruit Rot Studies in Puerto Rico

This review focuses on work in Puerto Rico, although there is little awareness of CFR there. Many coffee farmers in Puerto Rico attribute anthracnose symptoms to sunburn (which is abiotic) and do not realize there is a biotic component.

However, recent studies of CFR in Puerto Rico have revealed several novel aspects of the disease; they probably occur elsewhere as well, although parallel studies have not yet been conducted in other countries. First, four species of *Colletotrichum* were isolated from CFR lesions and shown to be pathogens using inoculation tests on green coffee fruits [21]. Two of these species, *C. theobromicola* and *C. tropicale*, had not previously been identified as pathogens of coffee fruits. Second, internal rot was observed in coffee fruits that were externally healthy; these internal rots were not mentioned previously in the literature. *C. tropicale* caused more external than internal rot, whereas *C. theobromicola* caused both, suggesting a disease complex of pathogens with different but overlapping niches. Recent theoretical work supports this example of niche differentiation among closely related species sharing common resources [22].

Third, fruits with sporulating colonies of the fungus *Beauveria bassiana* had significantly less fruit rot than fruits without [21]. This suggests there may be competition between *B. bassiana* and *Colletotrichum*, which was confirmed by inoculation experiments on green coffee fruits, both in the lab and in the field. Fourth, when fruits were inoculated with conidia of *Colletotrichum* spp., fruits with CBB entry holes developed significantly more rot than fruits without; the holes helped the pathogens to bypass the cuticle and enter the fruit. Fifth, CBBs inoculated with conidia of *Colletotrichum* and placed on bagged coffee branches in the field resulted in significantly more fruit rot than in control branches exposed to uninoculated CBBs. This demonstrated that the CBB can be a dispersal agent of coffee fruit rot pathogens. These connections between coffee fruit rot and the CBB (and also Bb) were novel; none had been shown previously.

Fruits with CBB holes had significantly more rot than unbored fruits, both external and internal [5]. However, the CBB is only one factor of many in CFR. In Puerto Rico, CFR is significantly more common at low altitudes [5] whereas the CBB infestation is significantly more common at high altitudes [23].

Another fungal genus, *Fusarium*, is also an important cause of CFR, though until recently studies have focused almost entirely on *Colletotrichum*. *Colletotrichum* was isolated more frequently from fruits in the early stages of external rot; *Fusarium* was isolated more frequently from fruits with advanced stages of external rots and from internal rots [5]. Five *Fusarium* species caused disease of green coffee fruits in inoculation tests, four of which had not previously been reported from coffee fruits [6]. Three of these species were also isolated from CBBs in rotted fruits, along with two other *Fusarium* species. When CBBs were inoculated with *Fusarium* conidia and placed on bagged coffee branches they bored holes in the fruits, and these fruits developed significantly more rot than fruits without CBBs; the CBB damage was associated with both external and internal rot [6]. *F. bostrycoides* (in the *F. solani* species complex) was the species most frequently isolated from CBBs and was associated with both external and internal rots.

These results make clear that (1) the CBB is a vector of *Fusarium*, and that its bore holes facilitate the entry of pathogens into coffee fruit; (2) the CBB contributes to both internal and external rots; (3) *Fusarium* is an overlooked factor in CFR; and (4) internal rots in externally healthy coffee fruits have been overlooked and can be serious.

The focus on Puerto Rico may seem excessive, but the relationship between CFR (including coffee berry disease) and the CBB, and the role of *Fusarium* in CFR, have not been studied elsewhere, though they probably exist elsewhere. In a survey of 16 papers on CFR (including coffee berry disease, and including many of the papers cited here), only one mentioned the CBB or *Fusarium*. That paper mentioned that the CBB could carry *Fusarium* as well as other fungi [24]. Similarly, very few papers on CBB mention CFR, unless they are citing the papers reviewed above (e.g., [2]). The primary purpose of this review is to bring these neglected relationships to the attention of CBB researchers.

The studies cited above have several implications for CFR and the CBB that they do not discuss. Four such implications are discussed below: production of volatiles, evidence of symbiosis between the CBB and *Fusarium*, interactions among trophic levels, and a possible role for bacteria. They are speculative in the sense that they propose experiments based on the literature rather than review experiments already published.

## 3. Volatiles

As stated above, CBB damage makes coffee fruits more susceptible to rot by fungi—but what about the other way around? We hypothesize that volatile compounds released during coffee fruit rot can attract CBBs, and that CBBs prefer to attack fruits that already have CBD. There is extensive literature on coffee fruit volatiles as attractants of CBBs, but very little is known about the CBB’s response to fungal or bacterial volatiles. Jaramillo et al. (2013) proposed:

“Noteworthy is the response of *H. hampei* to some of the compounds combined with ethanol and methanol. Because the biology of this insect suggests a close association with its host and its lineage with microorganisms, it would certainly gain from being able to detect fermenting odors since microbial infection of the berries during feeding would lead to the release of corresponding scent. As such, it is not surprising that *H. hampei* is attracted to the synthetic blend of a few of these VOCs combined with ethanol and methanol. Methanol and ethanol are products associated with wood decay and are components of the bouquet used by some ambrosia beetles for host detection acting synergistically with other attractive compounds in several species… [25].”

Fruit rot by microorganisms and the volatiles they produce has been assumed to repel herbivores [26]. In this view, microorganisms avoid competition for fruits with larger organisms by producing spoilage compounds that deter predation. However, in the case of citrus fruits, rot by the fungus *Penicillium digitatum* increased (rather than decreased) frugivory [27]. Since the CBB prefers to infest mature fruits, it is possible they use fruit rot-associated odors as cues.

Coffee fruit volatiles are of great interest for developing improved traps for CBBs. Currently, most traps use a methanol:ethanol mixture as an attractant but adding other fruit volatiles can help to attract the CBB and repel non-target insects more efficiently [28,29]. Volatiles emitted by *Colletotrichum*, *Fusarium*, and other coffee fruit rot-associated microorganisms should be tested as possible attractants of the CBB that could be added to these mixtures.

*Fusarium verticillioides* infects sugarcane, sometimes entering through holes bored by larvae of the moth *Diatraea saccharalis*. Fungal volatiles attract *D. saccharalis* which feeds on the plant [30]. Furthermore, *D. saccharalis* females not carrying the fungus prefer to oviposit on *F. verticillioides*-infected plants, while females carrying the fungus prefer to feed on uninfected plants. It would be interesting to see if the presence of fungi causes CBBs to alter host preference in a similar manner.

Chemical cues produced or modified by *Colletotrichum* and *Fusarium* might affect the CBB’s parasitoids or predators as well as the CBB itself [31]. For example, a parasitoid of the sugarcane borer *Diatraea saccharalis* preferred borer larvae from uninfected plants than from plants infected by the red rot fungus *F. verticillioides* [32]. Since feeding on red rot-infected plants decreased larval weight, its volatile compounds may have been a signal of lower resource quality to the parasitoid, or of the potential presence of mycotoxins in the larvae.

Moreover, *Colletotrichum* and *Fusarium* can produce metabolites in coffee beans that survive the roasting process and contribute bitterness, astringency, off-flavors, and decreased aroma to the final product [24]. Their impact on coffee quality needs to be established, especially since the studies cited above show that internal *Fusarium* rots are prevalent in coffee fruits and are not visible without cutting open the fruit; their impact on organoleptic quality of coffee is probably much greater than currently recognized.

## 4. Scolytid Beetles and *Fusarium*

There are also interesting, unresolved questions about the relationship between the CBB and *Fusarium*. *H. hampei* is in the family Curculionidae and subfamily Scolytinae. About half of the 7500 species of Scolytinae are ambrosia beetles that cultivate fungi in galleries in plant hosts [33]. This mutualism has evolved many times independently in the group [34]. Most ambrosia beetles farm fungi in the families Ophiostomataceae and Ceratocystidaceae; these fungi appear to have coevolved with the beetles and may not be able to reproduce and develop without them. However, a few ambrosia beetles cultivate *Fusarium* in the *F. solani* species complex [35].

Although *H. hampei* is not considered an ambrosia beetle, there is evidence that its relationship with *Fusarium* may be complex. *H. hampei* raised on coffee beans supplemented with *F. solani* produced more eggs and larvae than those without [36]. Replacing the fungus with ergosterol, a fungal sterol, also increased the CBB’s reproduction and survival. Setae on the pronotum, the first segment of the thorax, were effective at trapping and dispersing spores, effectively functioning as a type of mycetangium, or fungus-transporting structure. However, another nutritional experiment found no significant effect of *Fusarium* supplementation [37].

Morales-Ramos et al. propose a mutualistic relationship between the CBB and *F. solani*, but do not describe how the insect benefits the fungus [36]. That missing evidence can be found in Serrato-Diaz et al. [6]: the CBB transmits the fungus to coffee fruits where it propagates, causing internal rots. It is possible that this fruit rot in turn attracts more CBBs, as suggested above. However, sporulation followed by water dispersal is presumed to be the main source of infection [38]. If the CBB vectors *Fusarium* to the coffee fruit, *Fusarium* rots the fruit and provides important nutrients for the CBB and its larvae, and then *Fusarium* is dispersed to new fruits by the CBB, then this relationship has many similarities to the relationship between wood-boring scolytids and their fungal mutualists. On the other hand, there are also reports of *F. oxysporum* as an opportunistic pathogen of the CBB [39], and an unidentified *Fusarium* caused mortality on CBBs in lab experiments [40]. In general, tripartite symbioses among plants, plant pathogens, and herbivores are complex and variable in outcomes [41].

## 5. Trophic Interactions Involving CBBs and CFR

Ivette Perfecto and colleagues have studied interactions among organisms in coffee farms in Chiapas, Mexico [42]. Their models include fungal pathogens and insect pests of coffee, other fungi and insects that attack these pathogens and pests, and ants (Formicidae, especially *Azteca*) that have multiple interactions with several of the organisms involved. One of the conclusions is that the system is complex on several trophic levels, and a control measure that targets one organism may have unintended effects on others.

**Figure 2:**
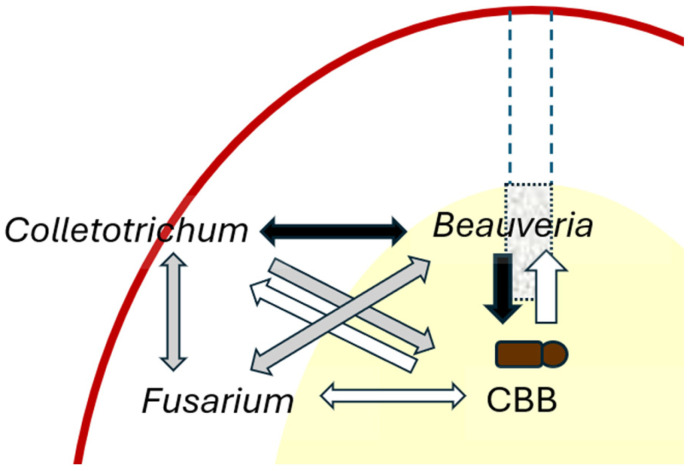
Possible interactions in a coffee fruit. Arrow color indicates interactions: clear, positive; black, negative; gray, unclear, no experimental evidence. The red line represents the coffee fruit exocarp, the yellow area is the developing seed, and the dashed lines show the CBB entry hole. *Beauveria* feeds on CBB, kills it, and competes with *Colletotrichum*. Its interaction with *Fusarium* is unknown. *Fusarium* contributes to the nutrition of the CBB; its relationship with *Colletotrichum* is unknown. The CBB is a vector of *Fusarium* and also facilitates the entry of *Colletotrichum*. Volatiles from *Colletotrichum* possibly attract the CBB to the rotting fruit. Half of the interactions have not been explored sufficiently to suggest whether they are negative or positive.

Based on the studies reviewed here, a similar model can be developed on the level of a single coffee fruit (Figure 2). The CBB attacks the coffee fruit directly and also indirectly by facilitating fruit rot. Bb attacks the CBB and also competes with the pathogen *Colletotrichum*, providing two levels of protection to the coffee fruit. Bb does not appear to compete efficiently with *Fusarium* [21].

Other interactions remain unexplored. First, how do *Fusarium* and *Colletotrichum* interact? Do they facilitate each other in degrading the fruit, as may be inferred from the fact that they generally colonize different parts of the fruit? Or are they competitors? Since several species of each genus are involved, and several species are more common in certain parts of the fruit and at certain stages of decay than in others [6,21], there are probably cases of facilitation and competition happening simultaneously. This implies a more complex model.

## 6. Hygiene, Control, and Overwintering

Another unexplored parallel between CFR and CBB is hygiene and control. There is extensive and widely known literature on the importance of hygiene for CBB control [43]. Hygiene should likewise be useful for control of CFR; that is, removal of infected fruits after harvest to reduce levels of inoculum. However, recent reviews and studies of CFR do not mention hygiene [9,44,45].

Adjusting harvest time to remove infested fruits could be an effective tool for CBB control, as shown by mathematical modelling of CBB populations; that is, when CBB damage is high an early harvest may prevent further cycles of infestation [46]. The same may be true of CFR, though no similar studies have been conducted.

In both cases it is not entirely clear where the pathogen reservoir is between seasons. In most places with a single harvest season per year, there is a period of several months when there are no coffee fruits sufficiently ripe to attack. Again, this has been extensively studied in the CBB, and some of the literature may be useful when applied to CFR. The CBB has a strong preference for coffee fruits over other potential hosts, so its whereabouts when no coffee fruits are available has been debated for over a century [47]. CFR pathogens are apparently not host specific [10], but it is still important to identify reservoirs where the pathogens survive when no coffee fruits are available.

## 7. Bacteria as Potential Pathogens and Biocontrol Agents

Figure 2 does not include bacteria. It is reasonable to assume that bacteria participate in coffee berry disease and coffee fruit rot; rotted fruits contain bacteria as well as fungi. Are these bacteria primary pathogens, or are they saprotrophs or secondary pathogens of already-rotted tissue? Bacteria have been tested as potential biological control agents against CBD, with promising results [48], but their role as pathogens in the disease has apparently not been tested. Two reviews on CBD do not mention bacteria as possible pathogens [9,49]. Studies on the bacterial microbiota of the CBB have mainly focused on caffeine degradation [50], cellulose metabolism [51], metabolism in general and biological control [52], and sex ratio [53,54]; bacteria as potential pathogens have been overlooked.

There is, however, evidence that bacteria can help protect coffee fruits from CBD. In experiments in Kenya, coffee fruits with the richest microbiota (including bacteria, yeasts, and filamentous fungi) had lower levels of CBD than fruits in areas with less microbiota [55]. Fungicide application decreased microbiota and increased CBD. Fungi isolated from coffee fruits in Kenya inhibited *C. kahawae* growth in vitro [56]; it is likely that some bacteria could have had a similar protective effect. Fungicides that decrease Bb populations may lead to larger CBB populations, which could partly explain the increase in CBD noted.

## 8. Implications for CBB Management and Future Studies

The above discussion suggests complex interactions, many of which are still unstudied, but it has some simple implications for management. First, controlling CBB will have a secondary benefit of reducing CFR [6,21]. Application of Bb early in the season should help to control both. Conversely, controlling CFR could possibly help reduce CBB damage if microbial volatiles serve as CBB attractants.

In Puerto Rico, most coffee growers have stopped trying to control the CBB. In the first ten years after its arrival in 2007, many farms used alcohol traps and applied Mycotrol^®^, a formulation of Bb [23]. More recently, neither of these control measures is common due to a lack of farm labor and capital; the focus has been on recovery from Hurricane Maria in 2017. Evidence that controlling the CBB can also help control CFR, and that Bb may control both, may provide an incentive to resume the use of control measures.

Fears that *C. kahawae* may spread from Africa to other areas [17,57] may be assuaged by the evidence that other fungi are already occupying the same niche.

Volatiles produced by *Colletotrichum* and *Fusarium* should be tested as possible additions to CBB traps.

Use of fungicides to control CFR/CBD may be counterproductive, by inhibiting the microbiota that competes with the pathogens on the surface of the fruit, and by killing Bb, a pathogen of the CBB whose damage makes fruits susceptible to CFR.

The studies cited above contradict and expand the prevailing concept of the disease in several ways: they showed an association between CFR and the CBB, that CFR attack on green fruits is not limited to Africa nor to high elevations, that *Fusarium* is an important pathogen of CFR, and that internal fruit rot, invisible from the outside, is a serious aspect of the disease.

## Abbreviations

Bb: *Beauveria bassiana*; CBB: coffee berry borer; CBD: coffee berry disease; CFR: coffee fruit rot.

## References

[1] Gaitán A.L., Cristancho M.A., Castro Caicedo B.L., Rivillas C.A., Cadena Gómez G. (2015). Compendium of Coffee Diseases and Pests.

[2] Johnson M.A., Ruiz-Diaz C.P., Manoukis N.C., Rodrigues J.C.V. (2020). Coffee Berry Borer (*Hypothenemus hampei*), a Global Pest of Coffee: Perspectives from Historical and Recent Invasions, and Future Priorities. Insects.

[3] Macdonald J. (1926). A preliminary account of a disease of green coffee berries in Kenya Colony. Trans. Br. Mycol. Soc..

[4] Rayner R.W. (1952). Coffee berry disease—A survey of investigations carried out up to 1950. East Afr. Agric. J..

[5] Caldwell M.Y., Mariño Y.A., Medina A.G., Serrato-Díaz L.M., Bayman P. (2023). Coffee fruit rot in Puerto Rico: Distribution, ecology and associated fungi. Eur. J. Plant Pathol..

[6] Serrato-Diaz L.M., Mariño Y.A., González J.D.J., Goenaga R., Bayman P. (2024). Coffee Fruit Rot: The previously unrecognized role of *Fusarium* and its interactions with the coffee berry borer (*Hypothenemus hampei*). Phytopathology.

[7] Nutman F.J., Roberts F.M. (1960). Investigations on a disease of *Coffea arabica* caused by a form of *Colletotrichum coffeanum* Noack: II. Some factors affecting germination and infection, and their relation to disease distribution. Trans. Br. Mycol. Soc..

[8] Griffiths E., Gibbs J.N., Waller J.M. (1971). Control of coffee berry disease. Ann. Appl. Biol..

[9] Adugna G. (2024). Coffee berry disease: A century-old anthracnose of green berries of Arabica coffee (*Coffea arabica* L.) in Africa. J. Plant Dis. Prot..

[10] Batista D., Silva D.N., Vieira A., Cabral A., Pires A.S., Loureiro A., Guerra-Guimarães L., Pereira A.P., Azinheira H., Talhinhas P. (2017). Legitimacy and implications of reducing *Colletotrichum kahawae* to subspecies in plant pathology. Front. Plant Sci..

[11] Wallis J.A.N. (1965). Coffee Berry Disease—1964. Pest Artic. News Summ. Sect. B Plant Dis. Control.

[12] Bridge P.D., Waller J.M., Davies D., Buddie A.G. (2008). Variability of *Colletotrichum kahawae* in relation to other Colletotrichum species from tropical perennial crops and the development of diagnostic techniques. J. Phytopathol..

[13] Cabral A., Azinheira H.G., Talhinhas P., Batista D., Ramos A.P., Silva M.D.C., Oliveira H., Várzea V. (2020). Pathological, morphological, cytogenomic, biochemical and molecular data support the distinction between *Colletotrichum cigarro* comb. et stat. nov. and Colletotrichum kahawae. Plants.

[14] Waller J.M., Bridge P.D., Black R., Hakiza G. (1993). Characterization of the coffee berry disease pathogen, *Colletotrichum kahawae* sp. nov. Mycol. Res..

[15] Weir B.S., Johnston P.R., Damm U. (2012). The *Colletotrichum gloeosporioides* species complex. Stud. Mycol..

[16] Silva D.N., Talhinhas P., Cai L., Manuel L., Gichuru E.K., Loureiro A., Várzea V., Paulo O.S., Batista D. (2012). Host-jump drives rapid and recent ecological speciation of the emergent fungal pathogen *Colletotrichum kahawae*. Mol. Ecol..

[17] The Australia Group (2022). List of Plant Pathogens for Export Control. https://www.dfat.gov.au/publications/minisite/theaustraliagroupnet/site/en/plants.html.

[18] Ferrucho R.L., Marín-Ramírez G.A., Ochoa-Corona F., Ángel C.A. (2024). PCR-based detection for the quarantine fungus *Colletotrichum kahawae*, a biosecurity threat to the coffee (*Coffea arabica*) industry worldwide. Plant Dis..

[19] Pardo-De la Hoz C.J., Calderón C., Rincón A.M., Cárdenas M., Danies G., López-Kleine L., Restrepo S., Jiménez P. (2016). Species from the *Colletotrichum acutatum*, *Colletotrichum boninense* and *Colletotrichum gloeosporioides* species complexes associated with tree tomato and mango crops in Colombia. Plant Pathol..

[20] Rojas P., Pardo-De la Hoz C.J., Calderón C., Vargas N., Cabrera L.A., Restrepo S., Jiménez P. (2018). First report of *Colletotrichum kahawae* subsp. *ciggaro* causing anthracnose disease on tree tomato in Cundinamarca, Colombia. Plant Dis..

[21] Serrato-Diaz L.M., Mariño Y.A., Bayman P. (2020). Pathogens causing anthracnose and fruit rots of coffee associated with the coffee berry borer and the entomopathogenic fungus *Beauveria bassiana* in Puerto Rico. Phytopathology.

[22] Hähn G.J., Damasceno G., Alvarez-Davila E., Aubin I., Bauters M., Bergmeier E., Biurrun I., Bjorkman A.D., Bonari G., Botta-Dukát Z. (2025). Global decoupling of functional and phylogenetic diversity in plant communities. Nat. Ecol. Evol..

[23] Mariño Y.A., Vega V.J., García J.M., Verle Rodrigues J.C., García N.M., Bayman P. (2017). The coffee berry borer (Coleoptera: Curculionidae) in Puerto Rico: Distribution, infestation, and population per fruit. J. Insect Sci..

[24] Ribeyre F., Avelino J., Oberthür T., Läderach P., Pohlan H.A.J., Cock J.H. (2012). Impact of field pests and diseases on coffee quality. Specialty Coffee: Managing Quality.

[25] Jaramillo J., Torto B., Mwenda D., Troeger A., Borgemeister C., Poehling H.M., Francke W. (2013). Coffee berry borer joins bark beetles in coffee klatch. PLoS ONE.

[26] Janzen D.H. (1977). Why fruits rot, seeds mold, and meat spoils. Am. Nat..

[27] Peris J.E., Rodríguez A., Peña L., Fedriani J.M. (2017). Fungal infestation boosts fruit aroma and fruit removal by mammals and birds. Sci. Rep..

[28] de la Rosa-Cancino W., Malo E.A., Gómez J., Valle-Mora J.F., Barrera J.F., Rojas J.C. (2023). Testing what we know about coffee volatiles affecting behaviour of *Hypothenemus hampei*. J. Appl. Entomol..

[29] Ruiz-Diaz C.P., Rodrigues J.C.V., Miro-Rivera E., Diaz-Vazquez L.M. (2023). Impact of the coffee berry borer on the volatile and semi-volatile compounds; qualitative profile of *Coffea arabica* berries. Food Chem. Adv..

[30] Franco F.P., Túler A.C., Gallan D.Z., Gonçalves F.G., Favaris A.P., Peñaflor M.F.G., Leal W.S., Moura D.S., Bento J.M.S., Silva-Filho M.C. (2021). Fungal phytopathogen modulates plant and insect responses to promote its dissemination. ISME J..

[31] Thomas G., Rusman Q., Morrison W.R., Magalhães D.M., Dowell J.A., Ngumbi E., Osei-Owusu J., Kansman J., Gaffke A., Pagadala Damodaram K.J. (2023). Deciphering plant-insect-microorganism signals for sustainable crop production. Biomolecules.

[32] Peñaflor M.F.G., Bento J.M.S. (2019). Red-rot infection in sugarcane attenuates the attractiveness of sugarcane borer-induced plant volatiles to parasitoid. Arthropod-Plant Interact..

[33] Mueller U.G., Gerardo N.M., Aanen D.K., Six D.L., Schultz T.R. (2005). The evolution of agriculture in insects. Annu. Rev. Ecol. Evol. Syst..

[34] Mayers C.G., Harrington T.C., Masuya H., Jordal B.H., McNew D.L., Shih H.H., Roets F., Kietzka G.J. (2020). Patterns of coevolution between ambrosia beetle mycangia and the Ceratocystidaceae, with five new fungal genera and seven new species. Persoonia.

[35] Kasson M.T., O’Donnell K., Rooney A.P., Sink S., Ploetz R.C., Ploetz J.N., Konkol J.L., Carrillo D., Freeman S., Mendel Z. (2013). An inordinate fondness for *Fusarium*: Phylogenetic diversity of fusaria cultivated by ambrosia beetles in the genus *Euwallacea* on avocado and other plant hosts. Fungal Genet. Biol..

[36] Morales-Ramos J.A., Rojas M.G., Sittertz-Bhatkar H., Saldaña G. (2000). Symbiotic relationship between *Hypothenemus hampei* (Coleoptera: Scolytidae) and *Fusarium solani* (Moniliales: Tuberculariaceae). Ann. Entomol. Soc. Am..

[37] Pérez J., Infante F., Vega F.E. (2007). Microorganismos asociados a la broca del café: ¿existe realmente un mutualismo?. Proc. Manejo Broca-do-Café.

[38] Bragard C., Baptista P., Chatzivassiliou E., Di Serio F., Gonthier P., Jaques Miret J.A., Justesen A.F., MacLeod A., Magnusson C.S. (2022). Pest categorisation of *Fusarium oxysporum* f. sp. *cubense* Tropical Race 4. EFSA J..

[39] Bustillo A.E., Cárdenas R., Posada F.J. (2002). Natural enemies and competitors of *Hypothenemus hampei* (Ferrari)(Coleoptera: Scolytidae) in Colombia. Neotrop. Entomol..

[40] Pérez-López E.J., Posada-Flórez F.J., González-García M.T. (1996). 1996. Patogenicidad de un aislamiento de *Fusarium* sp. encontrado infectando la broca del café, *Hypothenemus hampei*. Rev. Colomb. Entomol..

[41] Li Y., Duan T., Li Y. (2021). Research progress in the interactions of fungal pathogens and insect pests during host plant colonization. J. Plant Dis. Protect..

[42] Perfecto I., Vandermeer J. (2015). Coffee Agroecology: A New Approach to Understanding Agricultural Biodiversity, Ecosystem Services and Sustainable Development.

[43] Benavides P., Bustillo A.E., Cárdenas R., Montoya E.C. (2003). Análisis biológico y económico del manejo integrado de la broca del café en Colombia. Cenicafé.

[44] Giddisa G. (2016). A review on the status of coffee berry disease (*Colletotrichum kahawae*) in Ethiopia. J. Biol. Agric. Healthc..

[45] Motisi N., Ribeyre F., Poggi S. (2019). Coffee tree architecture and its interactions with microclimates drive the dynamics of coffee berry disease in coffee trees. Sci. Rep..

[46] Marcano M., Bose A., Bayman P. (2021). A one-dimensional map to study multi-seasonal coffee infestation by the coffee berry borer. Math. Biosci..

[47] Vega V.J., Mariño Y.A., Deynes D., Greco E.B., Bright D.E., Bayman P. (2020). A beetle in a haystack: Are there alternate hosts of the coffee berry borer (*Hypothenemus hampei*) in Puerto Rico?. Agronomy.

[48] Alemu K., Adugna G., Lemessa F., Muleta D. (2023). Biocontrol potentials of native bacterial strains for the management of coffee berry disease (*Colletotrichum kahawae*) in Ethiopia. Biocontrol Sci. Technol..

[49] Alemu K., Adugna G., Lemessa F., Muleta D. (2016). Current status of coffee berry disease (*Colletotrichum kahawae* Waller & Bridge) in Ethiopia. Arch. Phytopath. Plant Prot..

[50] Ceja-Navarro J.A., Vega F.E., Karaoz U., Hao Z., Jenkins S., Lim H.C., Kosina P., Infante F., Northen T.R., Brodie E.L. (2015). Gut microbiota mediate caffeine detoxification in the primary insect pest of coffee. Nat. Commun..

[51] Azizah A., Purwatiningsih P., Wiyono H.T., Muzakhar K. Morphological and biochemical characteristic of endosymbiont cellulolytic bacteria from gut of *Hypothenemus hampei* Ferr. and its enzyme activity. Proceedings of the AIP Conference Proceedings 2020.

[52] Mejía-Alvarado F.S., Ghneim-Herrera T., Góngora C.E., Benavides P., Navarro-Escalante L. (2021). Structure and dynamics of the gut bacterial community across the developmental stages of the coffee berry borer, *Hypothenemus hampei*. Front. Microbiol..

[53] Mariño Y.A., Verle Rodrigues J.C., Bayman P. (2017). *Wolbachia* affects reproduction and population dynamics of the coffee berry borer (*Hypothenemus hampei*): Implications for biological control. Insects.

[54] Mariño Y.A., Ospina O.E., Verle Rodrigues J.C., Bayman P. (2018). High diversity and variability in the bacterial microbiota of the coffee berry borer (Coleoptera: Curculionidae), with emphasis on *Wolbachia*. J. Appl. Microbiol..

[55] Waller J.M., Masaba D.M. (2006). The microflora of coffee surfaces and relationships to coffee berry disease. Int. J. Pest Manag..

[56] Msenya H.N. (2024). Distribution and Diversity of Fungi and Their Biocontrol Potential in Managing Coffee Berry Disease in Kirinyaga County Kenya. Ph.D. Thesis.

[57] Vieira A., Silva D.N., Várzea V., Paulo O.S., Batista D. (2019). Genome-wide signatures of selection in *Colletotrichum kahawae* reveal candidate genes potentially involved in pathogenicity and aggressiveness. Front. Microbiol..

